# Dietary oxalate to calcium ratio and incident cardiovascular events: a 10-year follow-up among an Asian population

**DOI:** 10.1186/s12937-022-00773-1

**Published:** 2022-03-28

**Authors:** Zahra Bahadoran, Parvin Mirmiran, Fereidoun Azizi

**Affiliations:** 1 Nutrition and Endocrine Research Center, Research Institute for Endocrine Sciences, Shahid Beheshti University of Medical Sciences, Tehran, Iran; 2 Department of Clinical Nutrition and Dietetics, Faculty of Nutrition and Food Technology, National Nutrition and Food Technology Research Institute, Shahid Beheshti University of Medical Sciences, No. 24, Shahid-Erabi St., Yeman St., Velenjak, Tehran, Iran; 3 Endocrine Research Center, Research Institute for Endocrine Sciences, Shahid Beheshti University of Medical Sciences, Tehran, Iran

**Keywords:** Oxalate, Calcium, Cardiovascular disease

## Abstract

**Background and aim:**

The potential cardiovascular impact of usual intakes of oxalate (Ox) is uninvestigated. We evaluated the effect of dietary Ox and its interaction with dietary calcium (Ca) on incident cardiovascular disease (CVD).

**Methods:**

We included 2966 adult men and women aged 19–84 y without known CVD during baseline enrollment (2006–2008) of the Tehran Lipid and Glucose Study. Dietary intakes were assessed using a validated FFQ, and incident CVD (i.e., coronary heart disease, stroke, and CVD mortality) were documented through March 2018.

**Results:**

A 7.1% incident of CVD occurred during a median follow-up of 10.6 y. After multivariable adjustment for traditional risk factors and key dietary nutrients, including total fat and fiber, Ox intakes ≥220 mg/d increased incident CVD (HR T3 vs. T1 = 1.47, 95% CI = 1.02–2.12). This association was potentiated (HR T3 vs. T1 = 2.42, 95% CI = 1.19–4.89) in subjects who had a lower intake of Ca (< 981 mg/d); in a low-Ca diet, an even lower amount of dietary Ox (second tertile, 148–220 mg/d) was related to increased CVD events by 92% (HR = 1.92, 95% CI = 1.00–3.70). No association was observed between dietary Ox and CVD events in the presence of medium- and high levels of Ca intakes. The critical cut-off point of Ox-to-Ca for predicting CVD events was 0.14, which was related to an increased risk of CVD by 37% (HR = 1.37, 95% CI = 1.02–1.84).

**Conclusion:**

Higher dietary Ox intake appeared to be associated with a modestly elevated risk of incident CVD, especially in a diet with a lower amount of Ca.

## Introduction

A high-oxalate (Ox) diet is the leading cause of secondary hyperoxaluria (i.e., urinary Ox over 45–50 mg/24 h) [1, 2], a potential risk factor for systemic oxalosis and cardiovascular dysfunction [3]. High-Ox levels may exert atherogenic effects by induction of oxidative stress, inflammation, monocyte chemoattractant protein (MCP-1) secretion, or by elevating intracellular calcium in endothelial cells, preventing re-endothelialization and inducing endothelial cell toxicity [4–9]. Hyperoxalemia also leads to systemic inflammation and oxidative stress [10], predisposing to the development of cardiovascular disease (CVD). Increased endogenous production, from either excessive Ox content of the food or intestinal hyper-absorption, contributes to Ox accumulation in the body [1, 2].

Mean usual dietary intake of Ox has been reported in a range of 50–350 mg/d [11, 12]; however, it may surpass 1000–2000 mg/d when high Ox-rich foods are consumed [13, 14]. Urinary Ox is equally originated from both the endogenous (i.e., metabolism of glycine, glycolate, hydroxyproline, and ascorbic acid) and exogenous sources (i.e., Ox-containing foods, including spinach, leafy vegetables, legumes, nuts, beetroot, and cocoa) [1, 15]; however, dietary contribution may reach up to 80% [16, 17]. The amount of gastrointestinal (GI)-absorbed Ox is affected by the amount of dietary Ca; the dietary Ca-to-Ox ratio would be, therefore, theoretically associated with the risk of Ca-Ox kidney stones [16, 18, 19], an independent risk factor for the development of CVD [20, 21]. Decreased dietary Ca considerably increases the contribution of dietary Ox to urinary Ox excretion up to 50% [16]. A sufficient amount of dietary Ca can reduce Ox absorption and excretion, while an imbalanced dietary intake of Ca and Ox allows more soluble Ox to be absorbed in the GI tract and induced hyperoxaluria [18].

Considering the evidence addressing cardiovascular effects of elevated plasma Ox and hyperoxaluria, one can speculate that a high-Ox diet may increase CVD risk beyond its adverse effects on renal function. This hypothesis is not investigated within population-based settings. Furthermore, it is not clear whether dietary intakes of Ca-Ox can interact to modify the risk of CVD. Here, we evaluated cardiovascular effects of dietary Ox and its potential interaction with Ca intake levels in the Tehran Lipid and Glucose Study (TLGS), a large prospective cohort of an Asian population.

## Methods

### Study design and population

#### Study population

**Fig. 1:**
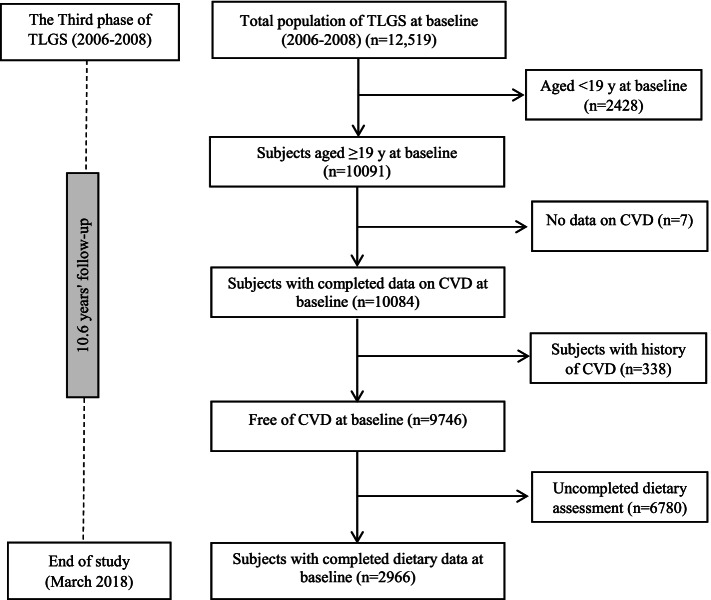
Flowchart of the study participants

Details of the TLGS design and study population have been reported extensively elsewhere [22]. In brief, the TLGS, begun in 1999, in a large-scale community-based prospective study on 15,005 individuals aged ≥ three years, a representative sample of residents of district 13 of Tehran, the capital city of Iran [22]. The measurements are repeated every three years to assess any change of non-communicable diseases (NCDs) risk. For the current study, we recruited 12,519 adults from both genders who had participated in the third TLGS phase (2006–2008). After excluding the participants under age 19 y, those who had a history of CVD or incomplete data regarding CVD, dietary intakes, demographics, anthropometric and biochemical measurements, 2966 healthy adults remained. Flowchart of the study population, recruited from the original sample of TLGS, is presented as Fig. 1. The remaining eligible participants were followed up to the end of the study (March 2018). The median follow-up period was 10.6 y (IQR = 9.9–11.1 y) from the baseline examination.

#### Dietary assessment

Information on dietary intake at baseline was collected using a validated 168-item food frequency questionnaire (FFQ). Details of dietary assessment have been reported elsewhere [23]. Reliability, relative validity [24], and stability of data retrieved by the FFQ over time [25] were confirmed. The Ox content of foods was derived from available documents measured and reported Ox concentration of different food items [13, 14, 26]. Estimated dietary Ox intakes were computed from the reported frequency of consumption of each specified unit of foods containing Ox. To remove confounding effect of under- or over-report of energy intakes on estimated-intakes of Ox, a residual adjustment was performed using a regression model (with total caloric intake as the independent variable and total Ox intake as the dependent variable).

#### Definition of outcomes and terms

The data collection of CVD outcomes has previously been described in detail by the TLGS research group [27, 28]. A specific outcome for each event was defined according to the international statistical classification of diseases and related health problems, 10th Revision CRITERIA, and the American heart association classification for cardiovascular events. Coronary heart disease (CHD)-related events included cases of definite myocardial infarction (MI) (diagnostic ECG and biomarkers), probable MI (positive ECG findings, cardiac symptoms or signs, and missing biomarkers, or positive ECG findings and equivocal biomarkers), unstable angina pectoris (new cardiac symptoms or changing symptom patterns and positive ECG findings with normal biomarkers), angiographic-confirmed CHD, and CHD death. Death from CHD or stroke was confirmed by reviewing the death certificate or medical records. Stroke was considered as a new neurological deficit that lasted at least 24 h. CVD was defined as any CHD-related event, stroke, or CVD death (definite fatal MI, definite fatal CHD, and definite fatal stroke) [27, 29].

Type 2 diabetes mellitus (T2DM) was defined as fasting serum glucose ≥126 mg/dL, 2-h serum glucose ≥200 mg/dL, or use of anti-diabetic medications [30]. Hypertension (HTN) was defined as systolic BP ≥ 140 mmHg, diastolic BP ≥ 90 mmHg, or current use of antihypertensive drugs [31].

#### Statistical analyses

Baseline characteristics of participants were described by mean ± standard deviation (SD) values, frequency (%), or median (inter-quartile range, IQR) across CVD status. Continuous variables with normal distribution were compared between the groups using independent t-test whereas dichotomous variables were compared using chi-square test. To compare variables with non-normal distributions, the Mann-Whitney U test was used.

We used Cox proportional hazard regression models to estimate hazard ratios (HRs) and 95% confidence interval (CI) of CVD events in relation to Ox intakes (as a log-transformed variable); the range of Ox intake across tertiles was < 148, 148–220 and > 220 with a median of 120, 181 and 277 mg/d. HRs (95% CI) were also estimated across low-, medium-, and high-Ca-diet, defined according to tertiles of Ca intakes as < 981, 981–1410 and > 1410 mg/d (with a median of 760, 1180, 1760 mg/d, respectively). Time to event was defined as time to end of follow-up (censored cases) or time to having an event, whichever occurred first. The proportional hazards assumption was tested. We censored participants at the time of death due to non-CVD causes, at the time of leaving the district, or study follow-up end time of March 2018.

A univariate analysis was performed to detect potential confounders (i.e. CVD risk score, total daily energy intake, and key nutrients), and the variables with a *P*_*E*_ (*P* value for entry) < 0.2 were included in the multivariable model. Three Cox models were conducted; model 1 was adjusted for CVD risk score (i.e., a sex-specific “general CVD” algorithms incorporating age, total cholesterol, HDL-C, SBP, treatment for HTN, smoking, and T2DM [32]. The TLGS research group reported the accuracy and clinical importance of the score among our population in detail [33]; using this score allowed us to account for major traditional CVD confounders without adding many variables and improving our models’ instability. Model 2 was further adjusted for eGFR as an indicator of renal function. The full model (model 3) additionally included daily energy intake and key nutrients including, fats and fiber. The median of each tertile was used as a continuous variable in the Cox proportional hazard regression models to estimate the overall trends of HRs across tertiles of Ox intake.

The receiver operator characteristic (ROC) curve analysis was used with an estimation of the variable sensitivity and specificity to determine the cut-off point of dietary Ox-to-Ca ratio for risk of developing CVD. The cut-off point was assessed by the maximum value of sensitivity + specificity – 1 (Youden index); the index is preferable for the finding of the optimal cut-off point because it is clinically translated to maximizing correct classification and minimizing misclassification rates [34].

All statistical analyses were performed using the Statistical Package for Social Science (version 20; IBM Corp., Armonk, NY, USA) and MedCalc Statistical Software version 15.8 (MedCalc Software bvba, Ostend, Belgium). A *P*-value < 0.05 being considered significant.

## Results

The median (IQR) of dietary Ox intake and mean (SD) Ox-to-Ca ratio was 182 (133–244 mg/d) and 0.17 (0.07). The baseline age of participants (44.8% men) was 39.4 ± 14.1 y. During a median follow-up of 10.6 y, 211 incident cases of CVD (7.1%). Baseline characteristics and distributions of the major known cardiometabolic risk factors across outcome status of the participants (with and without CVD events) are shown in Table 1. Compared with non-CVD subjects, those who developed CVD events were more likely to be older and had higher cardiometabolic risk factors (*P* for all < 0.01). Incident- compared to non-incident cases of CVD had higher intake of dietary Ox (191, IQR = 144–261 mg/d vs. 181, IQR = 132–243 mg/d) and higher Ox-to-Ca ratio (0.19 ± 0.08 vs. 0.16 ± 0.07).

**Table 1:** Baseline characteristics of the study participants (*n* = 2966)

	Participants with CVD outcome *(n = 211)*	Participants without CVD outcome *(n = 2755)*	*P* value
Age *(y)*	56.7 ± 10.9	38.1 ± 13.4	0.001
Male *(%)*	66.4	43.1	0.001
Smoking *(%)*	19.5	12.3	0.001
Body mass index *(m*^*2*^*/kg)*	28.6 ± 4.8	26.9 ± 4.9	0.001
Waist circumference *(cm)*	98.2 ± 11.3	88.9 ± 13.3	0.001
Systolic blood pressure *(mm Hg)*	127 ± 21	110 ± 16	0.001
Diastolic blood pressure *(mm Hg)*	79.9 ± 12.8	72.9 ± 10.5	0.001
Fasting blood glucose *(mg/dL)*	107 ± 37.9	90.2 ± 20.5	0.001
Serum triglycerides *(mg/dL)*^a^	166 (122–211)	117 (81–170)	0.001
HDL-C *(mg/dL)*	39.9 ± 9.2	42.9 ± 10.3	0.001
Serum Cr *(mg/dL)*	1.12 ± 0.21	1.04 ± 0.15	0.001
eGFR (*mL/min/1.73m*^*2*^*)*	66.8 ± 10.9	74.1 ± 11.8	0.001
Dietary oxalate *(mg/d)*^a^	191 (144–261)	181 (131–243)	0.016
Dietary calcium *(mg/d)*	1239 ± 560	1261 ± 542	0.564
Dietary oxalate-to-calcium ratio	0.19 ± 0.08	0.16 ± 0.07	0.004

Data are mean ± SD unless stated otherwise (independent t-test and chi-square test were used for continuous and dichotomous variables, respectively. To compare variables with non-normal distribution, Mann-Whitney U test was used

^a^Median (inter-quartile range, IQR)

Hazard ratios (95% CI) of CVD outcomes across tertile categories of dietary Ox intakes are reported in Table 2. In the fully adjusted Cox proportional hazards model, we observed a significantly elevated risk of CVD-related events in the highest, compared to the lowest tertile category (HR = 1.47, 95% CI = 1.02–2.12). This association was potentiated (HR _T3 vs. T1_ = 2.42, 95% CI = 1.19–4.89) in subjects who had a lower intake of Ca (< 981 mg/d); in a low-Ca diet, an even lower amount of dietary Ox (second tertile, 148–220 mg/d) was related to increased CVD events by 92% (HR = 1.92, 95% CI = 1.00–3.70). In the presence of medium- and high-levels of Ca intakes (range of 981–1410 and > 1410 mg/d, with a median of 1180 and 1760 mg/d, respectively), no association was observed between dietary Ox and CVD events.

**Table 2:** The hazard ratio (95% CI) of CVD events across tertile categories of dietary oxalate and dietary oxalate across different levels of dietary Ca (mg/d)^a^

	*Tertile1*	*Tertile2*	*Tertile 3*	*P for trend*
Dietary oxalate (median, mg/d)	148	181	277	
Case/total number	49/987	72/990	90/989	
*Crude*	1.00	1.46 (1.02–2.10)	1.86 (1.31–2.63)	0.002
*Model 1*	1.00	1.37 (0.95–1.97)	1.65 (1.16–2.34)	0.019
*Model 2*	1.00	1.37 (0.93–1.89)	1.60 (1.13–2.27)	0.030
*Model 3*	1.00	1.32 (0.91–1.90)	1.47 (1.02–2.12)	0.105
Dietary oxalate (mg/d)				
*Low-Ca diet*	1.00	1.92 (1.00–3.70)	2.42 (1.19–4.89)	0.046
*Medium-Ca diet*	1.00	1.18 (0.61–2.28)	1.34 (0.70–2.58)	0.668
*High-Ca diet*	1.00	1.29 (0.67–2.51)	1.66 (0.86–3.21)	0.306

Tertile 1 was considered as reference. Cox regression models were used. Model 1: Adjusted for CVD-risk score; Model 2: Additionally adjusted for eGFR; Model 3: Additionally adjusted for total energy intakes (kcal/d), dietary intakes of total fats (g/d), and fiber (g/d). Dietary oxalate was included in the models as a Log-transformed variable. Range of Ox intake across tertiles was < 148, 148–220 and > 220 with a median of 120, 181 and 277 mg/d

Low-, medium-, and high-Ca-diet were defined according to tertiles of Ca intakes as < 545, 545–981, and > 981 mg/d, with a median of 760, 1180, 1760 mg/d, respectively

^a^ Full model was only reported

Two sensitivity analyses of Cox regression were performed by excluding participants <30y or ≥ 65y at baseline, to consider potential confounding effect of different probability of CVD events and different dietary pattern among young and older-adults. Adjusted risk of CVD, among population older than 30y, increased by 27% (HR = 1.27, 95% CI = 0.88–1.82) and 47% (HR = 1.47, 95% CI = 1.03–2.10), in the second and third tertiles of Ox intakes. After exclusion of subjects older than 65y, adjusted risk of CVD was 1.33 (95% CI = 0.86–2.03) and 1.53 (1.00–2.35) in the second and third tertiles of Ox intakes.

**Fig. 2:**
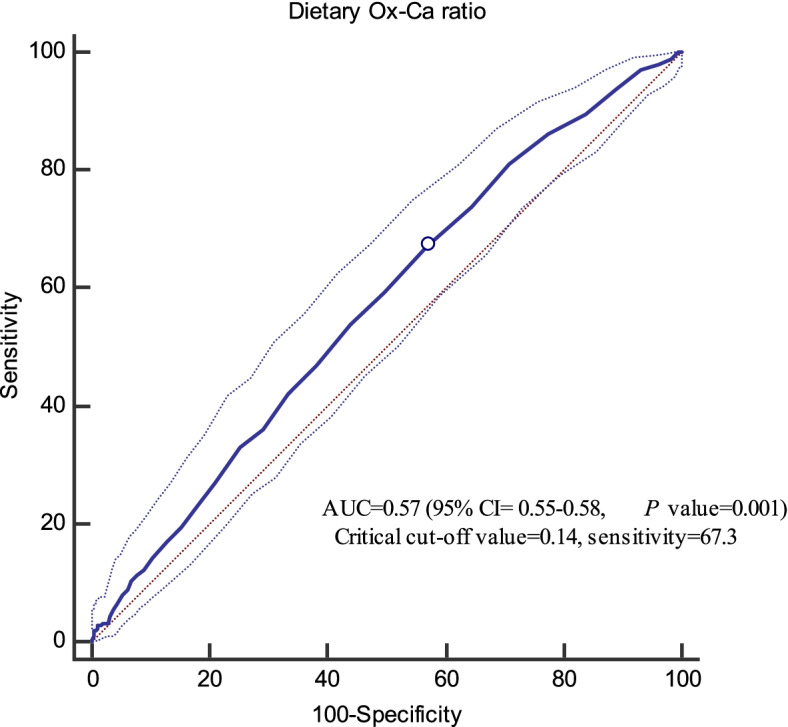
Cut-off point of Ox-to-Ca ratio for CVD events (0.14, sensitivity = 67.3%, Youden index = 0.10)

The critical cut-off value of the dietary Ox-to-Ca ratio for CVD events, as well as sensitivity, specificity, and AUC (*P*-value), are presented in Fig. 2. The critical cut-off point of Ox-to-Ca for predicting CVD events was 0.14 (The AUC and 95% CI = 0.57, 0.55–0.58, *P* value = 0.001; sensitivity = 67.3%). In the presence of traditional CVD risk factors, dietary Ox-to-Ca ratio higher than the cut-off value (≥ 0.14) was related to increased risk of CVD by 37% (HR = 1.37, 95% CI = 1.02–1.84).

## Discussion

In this prospective study of an Asian population, higher dietary Ox intake was associated with a 47% higher risk of incident CVD events independent of well-known confounders. In a low-Ca diet, the estimated CVD risk in relation to high-Ox intake (≥ 220 mg/d) was potentiated and reached to near 2.5-fold. Using a ROC curve analysis and calculating Youden index, a cut-off value of 0.14 was determined as a critical point of dietary Ox-to-Ca ratio (with an acceptable sensitivity ~ 70%); it means an imbalanced dietary intake of Ox and Ca indicated as ≥0.14, may be associated, both statistically and clinically, with an elevated risk of CVD.

To the best of our knowledge, this was the first population-based prospective study that evaluated the preliminary hypothesis regarding the potential cardiovascular effects of a high-Ox diet. Previously, studies indicated that a high-Ox diet might result in hyperoxaluria [1, 2] and increased risk of kidney failure, including both acute and chronic kidney disease, nephrolithiasis, and nephrocalcinosis [12, 35]. Evidence has also linked an imbalanced Ox-homeostasis with the development of CHD and stroke. Although a substantial causal relationship could not be established, some evidence implies that Ox accumulation in the human body (exhibited as increased plasma/urine Ox or oxalosis) may increase CVD risk [36].

The Ox-induced oxidative stress and inflammation [37, 38] and systemic oxalosis and deposition of Ox in cardiovascular tissues [39, 40] have been suggested as mechanisms that may connect oxalemia and hyperoxaluria with CVD events. A rich-Ox diet impacts monocyte cellular bioenergetics, mitochondrial complex activity, and inflammatory signaling in humans [41]. At cellular levels, exposure to high-Ox concentrations reduces glutathione levels, increases reactive oxygen species (ROS) generation, induces mitochondrial permeability transition mediated cell death, and MCP-1 secretion [4–7], events may lead to the development of vascular dysfunction. Elevated Ox levels may exert their atherogenic effects via increased intracellular calcium in endothelial cells and prevention of re-endothelialization [8]. Increased plasma Ox concentrations lead to accumulated vascular Ox levels, increases serum malondialdehyde (MDA), advanced oxidation protein products (AOPP), and tumor necrosis factor-α (TNF-α) levels and decreases superoxide dismutase activity [10].

The mean intake of dietary Ox in our population was ~ 200 mg/d, and grains-cereal products, spinach, green leafy vegetables, and nuts had more contribution to total Ox intakes. The estimated average daily Ox intake of the western population has been reported in a range of 44 and 351 mg/day (~ 0.5–4.0 mmol/day) [42]. The GI-absorption of Ox has been estimated to be 2.5–9% (a mean of 6.6% for a diet containing 180 mg Ox per 2500 kcal/d) [43, 44]. A linear relationship between dietary and urinary Ox has been reported (within dietary Ox ranged 50–750 mg/d) in a normal-Ca diet (~ 1000 mg/day) [15]. In the presence of a diet containing 250 mg Ox/day, decreasing ingested Ca by 60% (from 1002 to 391 mg), significantly increased contribution of dietary Ox in urinary Ox up to 53% [16]. A balanced Ox-Ca diet preventing secondary hyperoxaluria and its complications in healthy humans has not yet been defined. Considering the estimated critical point of 0.14 for Ox-to-Ca ratio in relation to CVD events and a population-mean of 200 mg/d Ox daily intake reported in different studies, a normal-Ca diet (~ 1200–1400 mg/day dietary Ca) may be recommended. Dietary Ca must be co-ingested with Ox-rich foods to maximize the Ox binding effect of Ca in the GI tract and decreased urinary Ox excretion [16].

Our study had some strengths, including its prospective design, relatively large sample size with long-term follow-up, detailed data on well-known risk factors, and potential confounders. The TLGS-nutrition cohort is also notable for its specifically designed and validated FFQ that was sensitive to evaluate usual dietary intakes of the Iranian population. Since the questionnaire included several foods with relatively high-Ox content, we could derive an accurate estimation of Ox, as it was comparable with mean Ox intake of other populations with relatively same dietary patterns. However, some researchers have argued that using semi-quantitative FFQ to estimate Ox intake is a limitation because many Ox-rich foods, including spinach, beets, and chocolate, mainly consumed on an irregular basis, are not adequately addressed in the questionnaire [42]. A specifically developed questionnaire for assessing Ox-rich foods and an accurate estimation of total Ox intake could help obtain more meaningful data.

Some limitations of our study also warrant discussion. First, as inherent into any prospective study, some degree of misclassification might have occurred, leading to biased estimated HRs towards the null because of potential changes in an individual’s diet, as well as changes in confounders during the study follow-up. Second, we have no data on plasma Ox levels and urinary Ox-Ca concentrations of the study participants; such data provides more insights into intestinal-renal handling of Ox and the status of Ox homeostasis in the body. Third, although major-known confounding variables were adjusted in our models, there may still be residual or unmeasured confounders that may result in biased exposure effect estimates. In our study, we could not assess genome-wide association to examine potential genetic variants in relation to Ox metabolism. Lacking data of potential polymorphisms of SCL26A6 (i.e., an anion exchanger expressed on the apical membrane in kidney and intestines, serving as the primary pathway for Ox absorption and secretion) [1, 45] may lead to an unbiased causal estimate of the cardiovascular effect of dietary Ox. Lacking SLC26A6 results in a defect in intestinal Ox secretion, enhanced net absorption of Ox, elevated plasma levels of Ox, and hyperoxaluria [46]. On the other hand, the potential role of gut microbiota and abundance of species handling Ox metabolism (e.g., *Oxalobacter formigenes*) and dietary habits influencing Ox degradation by the intestinal bacterial community [47, 48] did not consider in our analyses.

## Conclusion

A high-Ox diet, specifically in the presence of lower dietary Ca, may increase risk of developing CVD events. A balanced Ox-Ca needs to be considered, especially in subjects with a background of renal dysfunction, to prevent diet-induced secondary hyperoxaluria and oxalemia as a risk factor of cardiovascular dysfunction.

## Data Availability

Data will be presented upon forwarding the request to the corresponding author (mirmiran@endocrine.ac.ir) and confirmation of the director of RIES (azizi@endocrine.ac.ir).

## Abbreviations

AOPP: Advanced oxidation protein products
BP: Blood pressure
Ca: Calcium
CHD: Coronary heart disease
CI: Confidence interval
CVD: Cardiovascular disease
FFQ: Food frequency questionnaire
GI: Gastrointestinal
HR: Hazard ratio
HTN: Hypertension
IQR: Inter-quartile rage
MCP-1: Chemoattractant protein-1
MDA: Malondialdehyde
NCDs: Non-communicable diseases
Ox: Oxalate
ROC: Receiver operator characteristic
ROS: Reactive oxygen species
T2DM: Type 2 diabetes mellitus
TLGS: Tehran Lipid and Glucose Study
TNF-α: Tumor necrosis factor-α
